# Supplementary material for: Bulk and Single-Cell RNA Sequencing Elucidate the Etiology of Severe COVID-19
Source: Int J Mol Sci. 2024 Mar 14;25(6):3280. doi: 10.3390/ijms25063280 (PMC10970417; doi:10.3390/ijms25063280)
Supplement: Supplementary file 1 [file ijms-25-03280-s001.zip › ijms-2757125-supplementary.pdf]

Supplementary methods.

The review should be interested only in biologically well-interpreted gene expression datasets that made an impact on how we interpret the etiology of COVID-19. Therefore, the studies reviewed were selected based on the following criteria:

1. They were highly cited publications referencing well-defined, structured, and annotated high-throughput gene expression datasets.
2. They focused on the etiology of COVID that is on immune responses, inflammatory processes, and long-term tissue damage in a biomedically informative manner. I excluded datasets that focused on therapy or strain detection.
3. The authors submitted their datasets to online bioinformatics databases, making them available for future meta-analysis.
4. There was a well-described relevant methods section for genomics material.

The studies were identified through searches conducted on three databases: PubMed, Gene Expression Omnibus (GEO), and Elsevier's abstract and citation database (Scopus). PubMed was used to identify relevant publications, most of which had a dataset in GEO. Scopus was used to identify the most widely cited publications. Four keywords were used for these searches: COVID-19 and SARS-CoV-2, RNA-seq or scRNA-seq.

To access a GEO dataset, use the following formula:

https://www.ncbi.nlm.nih.gov/geo/query/acc.cgi?acc=GEOID

For example, to access a dataset linked with GSE147507, use the following link:

https://www.ncbi.nlm.nih.gov/geo/query/acc.cgi?acc=GSE147507

Moreover, the genomics studies chosen for review relied on standard NGS protocols and computational tools, including common software packages, standard workflows, and popular databases. Examples of computational methods relevant to bulk-sample RNA-seq are provided in Table S1. Additionally, methods relevant to scRNA-seq can be found in Table S2, while Table S3 contains links to the most pertinent bioinformatics resources.

The most common workflow involved parallel sequencing of polyadenylated mRNAs from experimental or survey samples. Subsequently, the NGS reads were aligned with a reference genome to calculate transcript abundance. Following this step, popular biostatistics software tools or packages were utilized to identify differentially expressed genes (DEGs). Finally, standard functional genomic tools and databases were employed to categorize the DEGs into functional classes.

GEO database entries adhere to the Minimum Information About a Next-generation Sequencing Experiment (MINSEQE) guidelines, which outline the minimum information that should be included when describing a sequencing study. The following elements are typically included:

- Raw data for each assay (e.g., FASTQ files)
- Final processed (normalized) data for the set of assays in the study (e.g., the gene expression data count matrix)
- Essential sample annotations (e.g., tissue, sex, and age) and the experimental factors and their values (e.g., compound and dose)
- Experimental design, including sample data relationships (e.g., which raw data file relates to which sample, technical/biological replicates)
- Sufficient annotation of the array or sequence features examined (e.g., gene identifiers, genomic coordinates)
- Essential laboratory and data processing protocols (e.g., which normalization method has been used to obtain the final processed data)

Table S1. Computational workflows for bulk-sample tissue- or cell-line RNA-seq.

Four illustrative examples were discussed in the table. Identifying differentially expressed genes (DEGs) between bulk samples was a common analysis goal. Once identified, DEGs were usually annotated to detect over-represented functional classes. The resulting data were then typically visualized using R/Bioconductor (BioC) or GraphPad software. Finally, the results were interpreted in biological terms.

Reference.	Bioinformatics pre-processing.	Statistical analyses.	Methods of annotation.	Visualization tools and methods.
[1].	Illumina's commercial software (*Alignment App*, and *Basespace*) was applied to perform quality control of reads, as well as their trimming, and alignment.	DEGs were identified using *DESeq2*. Sparse principal component analysis (*PCA*) was performed. Gene set enrichment (GSEA) analysis was also used.	DEGs were annotated using GO terms, as well as the protein-protein interaction networks functional enrichment analysis (*STRING*).	Heatmaps of gene expression levels were constructed using *heatmap.2* from R's *gplots* package. Volcano plots, dot plots, and scatterplots were constructed using *ggplot2*. Prism 8 (*GraphPad* Software, San Diego, CA, US) was employed to prepare some figures. A Venn diagram showed genes shared in each comparison. A dotplot was used to visualize enriched GO terms.
[2].	Reads were pre-processed applying Quality Control tool for High Throughput Sequence Data (*FastQC)*, trimmed with *Trimmomatic*, and then aligned with *STAR* or *bowtie2*. Read abundance was quantified using *Sambamba* and *FeatureCounts*.	*DESeq2* with false discovery rate (FDR) correction was applied to identify DEGs. Fisher's Exact Test was performed to calculate the overlap between transcriptomic signatures. Enrichment scores were calculated non-parametrically using a Kolmogorov Smirnoff-like random walk statistic. Simple linear regression was calculated in *GraphPad*.	Pathway analysis was performed using *Ingenuity* pathway analysis, as well as pathways from the Kyoto Encyclopedia of Genes and Genomes (*KEGG*). Enrichment in functional classes was also investigated using GO terms, as well as the Mouse	*GraphPad* Prism 8 was employed, as well as *ComplexHeatmap* from *BioC*. *Cytoscape* was utilized with the *clusterMaker2* plugin. R's packages *Monocle*, as well as *Adobe Illustrator* were also utilized to prepare figures.

			Genome Informatics (*MGI*) database.	
[3].	Paired-end sequencing was followed by sorting of reads with barcodes.	FDR correction for multiple testing was applied. Linear discriminant analysis was followed by Effect Size (LEfSe) calculations. Multiple testing correction was applied. Non-metric multidimensional scaling analysis was also performed. One-way *ANOVA* test with Tukey's *post-hoc* procedure was used to compare gene expression levels between samples.	Annotation of reads was achieved using *SALMON* with default parameters.	Prism 9 with *GraphPad* software was used to construct visualizations.

Table S2. Computational workflows for single-cell RNA sequencing (scRNA-seq).

Reference.	Bioinformatics pre-processing.	Statistical analyses.	Methods of annotation.	Visualization tools and methods.
[4]	*XPonent* software, *Luminex* v3.1. *FlowJo* v10. *TrimGalore* v0.4.4. *STAR* aligner v2.5.2b, *featureCounts* v1.5.2. Cell types were classified using signature tissue-specific genes using appropriate reference transcriptomes.	*DESeq2* v1.20.0, *ImpulseDE2* v1.4.0. *WGCNA* v1.69, *RnBeads*, *CellRanger*, *Seurat*, *SingleR*, *Monocle3*. *PCA* was performed to explore differences between healthy controls and COVID-19 patients.	*LOLA*, *tcR*, *GSEA*, *topGO*.	*WGCNA*, *Monocle3*, *Seurat*, *SingleR*, *Monocle3*. UMAP provided visualize cell-specificity in samples.
[5]	Reads were aligned, barcoded, and clustered using *CellRanger* v5.0. Quality control and exploratory analysis was performed using *Seurat* v3.2.3, *scrublet* v0.2.1, *CellBender* v0.2.0.	Statistical analyses were performed using *R* v4.0.2, *Python* 3.7.9, *SciPy* c1.4.1, *NumPy* v1.18.1, *Scanpy* v1.4.4.	*GSEA* was performed using *hypeR*, *CellPhoneDB* v2.0.0 was used to study cell-cell communication (*i.e.*, ligand-receptor interactions).	*R*, *ggplot2*, *GraphPad*, *UMAP* was used to visualize cell-type data with dimensionality reduction.
[6].	Reads were aligned using *CellRanger* software (10x Genomics). A cut-off value of 200 molecular identifiers used to select complex nuclei for further analysis. Cell free mRNA was removed using *SoupX*. Outliers and homotypic doublets were removed using Seurat and *DoubletFinder*. Barcoded but unmapped BAM reads were aligned using *STAR* to the SARS-	A *PCA* analysis was performed on *Seurat* objects to prepare *UMAP* diagrams. *Monocle3* was used to generate a pseudo-time trajectory analysis in a target cell type. General data analysis was performed using *R* and popular packaged such as	Biological pathway, and GO enrichment were performed using human data, and *Enrichr*, *Metascape*, or *GeneTrail* software. Ligand-receptor pairs were detected using different cell types using *CellChat* to detect cell-to-cell interactions (Secreted Signalling Pathways and	*UMAP* was used to cluster and visualize single-cell transcriptomic clusters. *R* and *ggplot2* were used to generate some figures.

	CoV-2 reference genome to detect viral transcripts (similarly to a *Viral-Track* pipeline).	*dplyr*, *patchwork*, or *BioC* packages.	human protein-protein interactions were used as a priori network information).	
[7].	*Cellbender* was used to remove background-level reads. Four clusters of cells were identified: conjunctival epithelium (superficial, basal, and suprabasal), and fibroblasts. Viral transcripts were also identified to find out which cell types can be infected with the virus.	DEGs were identified using a Seurat *FindMarkers* function (*P*-values were Bonferroni corrected). DEGs were identified between three experimental conditions: virus infected, exposed but uninfected, and unexposed. GSEA was calculated.	*Ingenuity* knowledge base was used to create mechanistic networks from lists of DEGs.	*UMAP* was used to cluster and visualize single-cell transcriptomic clusters.
[8].	Data aggregated using a *CellRanger* were analyzed by *Seurat*. The expression matrix was log-normalized in terms of unique molecular identifiers detected per cell.	A *PCA* analysis was performed. *PCA* dimensions from 1 through 4 were used to identify cell clusters on a uniform manifold approximation and projection (*UMAP*) diagram.	A single-sample gene-set variation analysis (*ssGSVA*) was used to identify gene sets differentially expressed in a target cell type.	*UMAP* — a statistical learning technique for dimension reduction in multivariate datasets — was used. Single-cell expression data were visualized on *UMAP* diagrams, violin plots, heat-maps, or volcano plots. *Nebulosa R* software package was also used.

Table S3. Other computational methods, tools, or resources.

These references provide valuable information for researchers interested in analyzing genomics data using various computational tools and methods.

Computational task.	Tool.	Literature reference.	Website.
Fast NGS read alignment.	STAR aligner.	[9].	https://github.com/alexdobin/STAR.
	Bowtie2.	[10].	https://bowtie-bio.sourceforge.net/bowtie2/index.shtml.
Quality control.	FastQC.	[11, 12].	https://www.bioinformatics.babraham.ac.uk/projects/fastqc.
Signal integration and deconvolution for scRNA-seq.	Seurat.	[13].	https://satijalab.org/seurat.
	Scanpy.	[14].	https://github.com/theislab/scanpy.
	UMAP.	[15-17].	https://umap-learn.readthedocs.io/en/latest.
Identification of DEGs.	DESeq2.	[18].	https://bioconductor.org/packages/release/bioc/html/DESeq2.html.
Annotation of DEGs.	GO database.	[19].	https://geneontology.org.
	KEGG pathways.	[20].	https://www.genome.jp/kegg/pathway.html.
	STRING.	[21].	https://string-db.org.
	CellPhoneDB.	[22].	https://www.cellphonedb.org.
Data visualization.	GraphPad.		https://www.graphpad.com.
	Ggplot2.	[23].	https://ggplot2.tidyverse.org.
General bioinformatics and scientific computing.	Bioconductor.	[24].	https://www.bioconductor.org.
	Python.	Cite individual packages.	https://www.python.org.
	NumPy.	[25].	https://numpy.org.
	SciPy.	[26].	https://scipy.org.
General statistics.	R.	Cite individual packages.	https://www.r-project.org.
Data sources.	Single-Cell Expression Atlas.	[27].	https://www.ebi.ac.uk/gxa/home.
	GEO.	[28].	https://www.ncbi.nlm.nih.gov/geo/.
	FANTOM5.	[29].	https://fantom.gsc.riken.jp/5/.

REFERENCES:

1. Blanco-Melo, D.; Nilsson-Payant, B. E.; Liu, W.-C.; Uhl, S.; Hoagland, D.; Muller, R.; Jordan, T. X.; Oishi, K.; Panis, M.; Sachs, D.; Wang, T. T.; Schwartz, R. E.; Lim, J. K.; Albrecht, R. A.; tenOever, B. R., Imbalanced Host Response to SARS-CoV-2 Drives Development of COVID-19. *Cell* **2020,** 181, (5), 1036-1045.e9.
2. Daamen, A. R.; Bachali, P.; Owen, K. A.; Kingsmore, K. M.; Hubbard, E. L.; Labonte, A. C.; Robl, R.; Shrotri, S.; Grammer, A. C.; Lipsky, P. E., Comprehensive transcriptomic analysis of COVID-19 blood, lung, and airway. *Scientific reports* **2021,** 11, (1), 7052-7052.
3. Brown, J. A.; Sanidad, K. Z.; Lucotti, S.; Lieber, C. M.; Cox, R. M.; Ananthanarayanan, A.; Basu, S.; Chen, J.; Shan, M.; Amir, M.; Schmidt, F.; Weisblum, Y.; Cioffi, M.; Li, T.; Rowdo, F. M.; Martin, M. L.; Guo, C.-J.; Lyssiotis, C. A.; Layden, B. T.; Dannenberg, A. J.; Bieniasz, P. D.; Lee, B.; Inohara, N.; Matei, I.; Plemper, R. K.; Zeng, M. Y., Gut microbiota-derived metabolites confer protection against SARS-CoV-2 infection. *Gut Microbes* **2022,** 14, (1), 2105609.
4. Bernardes, J. P.; Mishra, N.; Tran, F.; Bahmer, T.; Best, L.; Blase, J. I.; Bordoni, D.; Franzenburg, J.; Geisen, U.; Josephs-Spaulding, J.; K√∂hler, P.; K√ºnstner, A.; Rosati, E.; Aschenbrenner, A. C.; Bacher, P.; Baran, N.; Boysen, T.; Brandt, B.; Bruse, N.; D√∂rr, J.; Dr√§ger, A.; Elke, G.; Ellinghaus, D.; Fischer, J.; Forster, M.; Franke, A.; Franzenburg, S.; Frey, N.; Friedrichs, A.; Fu√ü, J.; Gl√ºck, A.; Hamm, J.; Hinrichsen, F.; Hoeppner, M. P.; Imm, S.; Junker, R.; Kaiser, S.; Kan, Y. H.; Knoll, R.; Lange, C.; Laue, G.; Lier, C.; Lindner, M.; Marinos, G.; Markewitz, R.; Nattermann, J.; Noth, R.; Pickkers, P.; Rabe, K. F.; Renz, A.; R√∂cken, C.; Rupp, J.; Schaffarzyk, A.; Scheffold, A.; Schulte-Schrepping, J.; Schunk, D.; Skowasch, D.; Ulas, T.; Wandinger, K. P.; Wittig, M.; Zimmermann, J.; Busch, H.; Hoyer, B. F.; Kaleta, C.; Heyckendorf, J.; Kox, M.; Rybniker, J.; Schreiber, S.; Schultze, J. L.; Rosenstiel, P., Longitudinal Multi-omics Analyses Identify Responses of Megakaryocytes, Erythroid Cells, and Plasmablasts as Hallmarks of Severe COVID-19. *Immunity* **2020,** 53, (6), 1296-1314 e9.
5. Melms, J. C.; Biermann, J.; Huang, H.; Wang, Y.; Nair, A.; Tagore, S.; Katsyv, I.; Rendeiro, A. F.; Amin, A. D.; Schapiro, D.; Frangieh, C. J.; Luoma, A. M.; Filliol, A.; Fang, Y.; Ravichandran, H.; Clausi, M. G.; Alba, G. A.; Rogava, M.; Chen, S. W.; Ho, P.; Montoro, D. T.; Kornberg, A. E.; Han, A. S.; Bakhoum, M. F.; Anandasabapathy, N.; Su√°rez-Fari√±as, M.; Bakhoum, S. F.; Bram, Y.; Borczuk, A.; Guo, X. V.; Lefkowitch, J. H.; Marboe, C.; Lagana, S. M.; Del Portillo, A.; Tsai, E. J.; Zorn, E.; Markowitz, G. S.; Schwabe, R. F.; Schwartz, R. E.; Elemento, O.; Saqi, A.; Hibshoosh, H.; Que, J.; Izar, B., A molecular single-cell lung atlas of lethal COVID-19. *Nature* **2021,** 595, (7865), 114-119.
6. Yang, A. C.; Kern, F.; Losada, P. M.; Agam, M. R.; Maat, C. A.; Schmartz, G. P.; Fehlmann, T.; Stein, J. A.; Schaum, N.; Lee, D. P.; Calcuttawala, K.; Vest, R. T.; Berdnik, D.; Lu, N.; Hahn, O.; Gate, D.; McNerney, M. W.; Channappa, D.; Cobos, I.; Ludwig, N.; Schulz-Schaeffer, W. J.; Keller, A.; Wyss-Coray, T., Dysregulation of brain and choroid plexus cell types in severe COVID-19. *Nature* **2021,** 595, (7868), 565-571.
7. Jackson, R. M.; Hatton, C. F.; Spegarova, J. S.; Georgiou, M.; Collin, J.; Stephenson, E.; Verdon, B.; Haq, I. J.; Hussain, R.; Coxhead, J. M.; Mudhar, H.-S.; Wagner, B.; Hasoon, M.; Davey, T.; Rooney, P.; Khan, C. M. A.; Ward, C.; Brodlie, M.; Haniffa, M.; Hambleton, S.; Armstrong, L.; Figueiredo, F.; Queen, R.; Duncan, C. J. A.; Lako, M.,

Conjunctival epithelial cells resist productive SARS-CoV-2 infection. *Stem Cell Reports* **2022,** 17, (7), 1699-1713.
8. Iwamura, C.; Hirahara, K.; Kiuchi, M.; Ikehara, S.; Azuma, K.; Shimada, T.; Kuriyama, S.; Ohki, S.; Yamamoto, E.; Inaba, Y.; Shiko, Y.; Aoki, A.; Kokubo, K.; Hirasawa, R.; Hishiya, T.; Tsuji, K.; Nagaoka, T.; Ishikawa, S.; Kojima, A.; Mito, H.; Hase, R.; Kasahara, Y.; Kuriyama, N.; Tsukamoto, T.; Nakamura, S.; Urushibara, T.; Kaneda, S.; Sakao, S.; Tobiume, M.; Suzuki, Y.; Tsujiwaki, M.; Kubo, T.; Hasegawa, T.; Nakase, H.; Nishida, O.; Takahashi, K.; Baba, K.; Iizumi, Y.; Okazaki, T.; Kimura, M. Y.; Yoshino, I.; Igari, H.; Nakajima, H.; Suzuki, T.; Hanaoka, H.; Nakada, T.-A.; Ikehara, Y.; Yokote, K.; Nakayama, T., Elevated Myl9 reflects the Myl9-containing microthrombi in SARS-CoV-2-induced lung exudative vasculitis and predicts COVID-19 severity. *Proceedings of the National Academy of Sciences of the United States of America* **2022,** 119, (33), e2203437119-e2203437119.
9. Dobin, A.; Davis, C. A.; Schlesinger, F.; Drenkow, J.; Zaleski, C.; Jha, S.; Batut, P.; Chaisson, M.; Gingeras, T. R., STAR: ultrafast universal RNA-seq aligner. *Bioinformatics* **2013,** 29, (1), 15-21.
10. Langmead, B.; Salzberg, S. L., Fast gapped-read alignment with Bowtie 2. *Nature Methods* **2012,** 9, (4), 357-359.
11. Ramirez-Gonzalez, R. H.; Leggett, R. M.; Waite, D.; Thanki, A.; Drou, N.; Caccamo, M.; Davey, R., StatsDB: platform-agnostic storage and understanding of next generation sequencing run metrics. *F1000Res* **2013,** 2, 248.
12. Kroll, K. W.; Mokaram, N. E.; Pelletier, A. R.; Frankhouser, D. E.; Westphal, M. S.; Stump, P. A.; Stump, C. L.; Bundschuh, R.; Blachly, J. S.; Yan, P., Quality Control for RNA-Seq (QuaCRS): An Integrated Quality Control Pipeline. *Cancer Inform* **2014,** 13, (Suppl 3), 7-14.
13. Hao, Y.; Hao, S.; Andersen-Nissen, E.; Mauck, W. M., 3rd; Zheng, S.; Butler, A.; Lee, M. J.; Wilk, A. J.; Darby, C.; Zager, M.; Hoffman, P.; Stoeckius, M.; Papalexi, E.; Mimitou, E. P.; Jain, J.; Srivastava, A.; Stuart, T.; Fleming, L. M.; Yeung, B.; Rogers, A. J.; McElrath, J. M.; Blish, C. A.; Gottardo, R.; Smibert, P.; Satija, R., Integrated analysis of multimodal single-cell data. *Cell* **2021,** 184, (13), 3573-3587 e29.
14. Wolf, F. A.; Angerer, P.; Theis, F. J., SCANPY: large-scale single-cell gene expression data analysis. *Genome Biol* **2018,** 19, (1), 15.
15. Satija, R.; Farrell, J. A.; Gennert, D.; Schier, A. F.; Regev, A., Spatial reconstruction of single-cell gene expression data. *Nature Biotechnology* **2015,** 33, (5), 495-502.
16. Butler, A.; Hoffman, P.; Smibert, P.; Papalexi, E.; Satija, R., Integrating single-cell transcriptomic data across different conditions, technologies, and species. *Nature Biotechnology* **2018,** 36, (5), 411-420.
17. Stuart, T.; Butler, A.; Hoffman, P.; Hafemeister, C.; Papalexi, E.; Mauck, W. M.; Hao, Y.; Stoeckius, M.; Smibert, P.; Satija, R., Comprehensive Integration of Single-Cell Data. *Cell* **2019,** 177, (7), 1888-1902.e21.
18. Love, M. I.; Huber, W.; Anders, S., Moderated estimation of fold change and dispersion for RNA-seq data with DESeq2. *Genome Biol* **2014,** 15, (12), 550-550.
19. Ashburner, M.; Ball, C. A.; Blake, J. A.; Botstein, D.; Butler, H.; Cherry, J. M.; Davis, A. P.; Dolinski, K.; Dwight, S. S.; Eppig, J. T.; Harris, M. A.; Hill, D. P.; Issel-Tarver, L.; Kasarskis, A.; Lewis, S.; Matese, J. C.; Richardson, J. E.; Ringwald, M.; Rubin, G. M.; Sherlock, G., Gene ontology: tool for the unification of biology. The Gene Ontology Consortium. *Nat Genet* **2000,** 25, (1), 25-9.

20. Kanehisa, M.; Goto, S.; Kawashima, S.; Okuno, Y.; Hattori, M., The KEGG resource for deciphering the genome. *Nucleic Acids Res* **2004,** 32, (Database issue), D277-80.
21. Szklarczyk, D.; Gable, A. L.; Lyon, D.; Junge, A.; Wyder, S.; Huerta-Cepas, J.; Simonovic, M.; Doncheva, N. T.; Morris, J. H.; Bork, P.; Jensen, L. J.; Mering, C. v., STRING v11: protein‚Äìprotein association networks with increased coverage, supporting functional discovery in genome-wide experimental datasets. *Nucleic Acids Res* **2019,** 47, (D1), D607-D613.
22. Efremova, M.; Vento-Tormo, M.; Teichmann, S. A.; Vento-Tormo, R., CellPhoneDB: inferring cell‚Äìcell communication from combined expression of multi-subunit ligand‚Äìreceptor complexes. *Nature Protocols* **2020,** 15, (4), 1484-1506.
23. Wickham, H., *ggplot2: elegant graphics for data analysis*. Springer: New York, 2009.
24. Gentleman, R. C.; Carey, V. J.; Bates, D. M.; Bolstad, B.; Dettling, M.; Dudoit, S.; Ellis, B.; Gautier, L.; Ge, Y.; Gentry, J.; Hornik, K.; Hothorn, T.; Huber, W.; Iacus, S.; Irizarry, R.; Leisch, F.; Li, C.; Maechler, M.; Rossini, A. J.; Sawitzki, G.; Smith, C.; Smyth, G.; Tierney, L.; Yang, J. Y.; Zhang, J., Bioconductor: open software development for computational biology and bioinformatics. *Genome biology* **2004,** 5, (10), R80.
25. Harris, C. R.; Millman, K. J.; van der Walt, S. f. J.; Gommers, R.; Virtanen, P.; Cournapeau, D.; Wieser, E.; Taylor, J.; Berg, S.; Smith, N. J.; Kern, R.; Picus, M.; Hoyer, S.; van Kerkwijk, M. H.; Brett, M.; Haldane, A.; del R√≠o, J. F. n.; Wiebe, M.; Peterson, P.; G√©rard-Marchant, P.; Sheppard, K.; Reddy, T.; Weckesser, W.; Abbasi, H.; Gohlke, C.; Oliphant, T. E., Array programming with NumPy. *Nature* **2020,** 585, (7825), 357-362.
26. Virtanen, P.; Gommers, R.; Oliphant, T. E.; Haberland, M.; Reddy, T.; Cournapeau, D.; Burovski, E.; Peterson, P.; Weckesser, W.; Bright, J.; van der Walt, S. J.; Brett, M.; Wilson, J.; Millman, K. J.; Mayorov, N.; Nelson, A. R. J.; Jones, E.; Kern, R.; Larson, E.; Carey, C. J.; Polat, ƒ.; Feng, Y.; Moore, E. W.; VanderPlas, J.; Laxalde, D.; Perktold, J.; Cimrman, R.; Henriksen, I.; Quintero, E. A.; Harris, C. R.; Archibald, A. M.; Ribeiro, A. H.; Pedregosa, F.; van Mulbregt, P., SciPy 1.0: fundamental algorithms for scientific computing in Python. *Nat Methods* **2020,** 17, (3), 261-272.
27. Papatheodorou, I.; Moreno, P.; Manning, J.; Fuentes, A. M.; George, N.; Fexova, S.; Fonseca, N. A.; Fullgrabe, A.; Green, M.; Huang, N.; Huerta, L.; Iqbal, H.; Jianu, M.; Mohammed, S.; Zhao, L.; Jarnuczak, A. F.; Jupp, S.; Marioni, J.; Meyer, K.; Petryszak, R.; Prada Medina, C. A.; Talavera-Lopez, C.; Teichmann, S.; Vizcaino, J. A.; Brazma, A., Expression Atlas update: from tissues to single cells. *Nucleic Acids Res* **2020,** 48, (D1), D77-D83.
28. Barrett, T.; Wilhite, S. E.; Ledoux, P.; Evangelista, C.; Kim, I. F.; Tomashevsky, M.; Marshall, K. A.; Phillippy, K. H.; Sherman, P. M.; Holko, M.; Yefanov, A.; Lee, H.; Zhang, N.; Robertson, C. L.; Serova, N.; Davis, S.; Soboleva, A., NCBI GEO: archive for functional genomics data sets--update. *Nucleic Acids Res* **2013,** 41, (Database issue), D991-5.
29. Consortium, F.; the, R. P.; Clst; Forrest, A. R.; Kawaji, H.; Rehli, M.; Baillie, J. K.; de Hoon, M. J.; Haberle, V.; Lassmann, T.; Kulakovskiy, I. V.; Lizio, M.; Itoh, M.; Andersson, R.; Mungall, C. J.; Meehan, T. F.; Schmeier, S.; Bertin, N.; Jorgensen, M.; Dimont, E.; Arner, E.; Schmidl, C.; Schaefer, U.; Medvedeva, Y. A.; Plessy, C.; Vitezic, M.; Severin, J.; Semple, C.; Ishizu, Y.; Young, R. S.; Francescatto, M.; Alam, I.; Albanese, D.; Altschuler, G. M.; Arakawa, T.; Archer, J. A.; Arner, P.; Babina, M.; Rennie, S.; Balwierz, P. J.; Beckhouse, A. G.; Pradhan-Bhatt, S.; Blake, J. A.;

Blumenthal, A.; Bodega, B.; Bonetti, A.; Briggs, J.; Brombacher, F.; Burroughs, A. M.; Califano, A.; Cannistraci, C. V.; Carbajo, D.; Chen, Y.; Chierici, M.; Ciani, Y.; Clevers, H. C.; Dalla, E.; Davis, C. A.; Detmar, M.; Diehl, A. D.; Dohi, T.; Drablos, F.; Edge, A. S.; Edinger, M.; Ekwall, K.; Endoh, M.; Enomoto, H.; Fagiolini, M.; Fairbairn, L.; Fang, H.; Farach-Carson, M. C.; Faulkner, G. J.; Favorov, A. V.; Fisher, M. E.; Frith, M. C.; Fujita, R.; Fukuda, S.; Furlanello, C.; Furino, M.; Furusawa, J.; Geijtenbeek, T. B.; Gibson, A. P.; Gingeras, T.; Goldowitz, D.; Gough, J.; Guhl, S.; Guler, R.; Gustincich, S.; Ha, T. J.; Hamaguchi, M.; Hara, M.; Harbers, M.; Harshbarger, J.; Hasegawa, A.; Hasegawa, Y.; Hashimoto, T.; Herlyn, M.; Hitchens, K. J.; Ho Sui, S. J.; Hofmann, O. M.; Hoof, I.; Hori, F.; Huminiecki, L.; Iida, K.; Ikawa, T.; Jankovic, B. R.; Jia, H.; Joshi, A.; Jurman, G.; Kaczkowski, B.; Kai, C.; Kaida, K.; Kaiho, A.; Kajiyama, K.; Kanamori-Katayama, M.; Kasianov, A. S.; Kasukawa, T.; Katayama, S.; Kato, S.; Kawaguchi, S.; Kawamoto, H.; Kawamura, Y. I.; Kawashima, T.; Kempfle, J. S.; Kenna, T. J.; Kere, J.; Khachigian, L. M.; Kitamura, T.; Klinken, S. P.; Knox, A. J.; Kojima, M.; Kojima, S.; Kondo, N.; Koseki, H.; Koyasu, S.; Krampitz, S.; Kubosaki, A.; Kwon, A. T.; Laros, J. F.; Lee, W.; Lennartsson, A.; Li, K.; Lilje, B.; Lipovich, L.; Mackay-Sim, A.; Manabe, R.; Mar, J. C.; Marchand, B.; Mathelier, A.; Mejhert, N.; Meynert, A.; Mizuno, Y.; de Lima Morais, D. A.; Morikawa, H.; Morimoto, M.; Moro, K.; Motakis, E.; Motohashi, H.; Mummery, C. L.; Murata, M.; Nagao-Sato, S.; Nakachi, Y.; Nakahara, F.; Nakamura, T.; Nakamura, Y.; Nakazato, K.; van Nimwegen, E.; Ninomiya, N.; Nishiyori, H.; Noma, S.; Noma, S.; Noazaki, T.; Ogishima, S.; Ohkura, N.; Ohimiya, H.; Ohno, H.; Ohshima, M.; Okada-Hatakeyama, M.; Okazaki, Y.; Orlando, V.; Ovchinnikov, D. A.; Pain, A.; Passier, R.; Patrikakis, M.; Persson, H.; Piazza, S.; Prendergast, J. G.; Rackham, O. J.; Ramilowski, J. A.; Rashid, M.; Ravasi, T.; Rizzu, P.; Roncador, M.; Roy, S.; Rye, M. B.; Saijyo, E.; Sajantila, A.; Saka, A.; Sakaguchi, S.; Sakai, M.; Sato, H.; Savvi, S.; Saxena, A.; Schneider, C.; Schultes, E. A.; Schulze-Tanzil, G. G.; Schwegmann, A.; Sengstag, T.; Sheng, G.; Shimoji, H.; Shimoni, Y.; Shin, J. W.; Simon, C.; Sugiyama, D.; Sugiyama, T.; Suzuki, M.; Suzuki, N.; Swoboda, R. K.; t Hoen, P. A.; Tagami, M.; Takahashi, N.; Takai, J.; Tanaka, H.; Tatsukawa, H.; Tatum, Z.; Thompson, M.; Toyodo, H.; Toyoda, T.; Valen, E.; van de Wetering, M.; van den Berg, L. M.; Verado, R.; Vijayan, D.; Vorontsov, I. E.; Wasserman, W. W.; Watanabe, S.; Wells, C. A.; Winteringham, L. N.; Wolvetang, E.; Wood, E. J.; Yamaguchi, Y.; Yamamoto, M.; Yoneda, M.; Yonekura, Y.; Yoshida, S.; Zabierowski, S. E.; Zhang, P. G.; Zhao, X.; Zucchelli, S.; Summers, K. M.; Suzuki, H.; Daub, C. O.; Kawai, J.; Heutink, P.; Hide, W.; Freeman, T. C.; Lenhard, B.; Bajic, V. B.; Taylor, M. S.; Makeev, V. J.; Sandelin, A.; Hume, D. A.; Carninci, P.; Hayashizaki, Y., A promoter-level mammalian expression atlas. *Nature* **2014,** 507, (7493), 462-70.
